# Mobile health clinic model in the COVID-19 pandemic: lessons learned and opportunities for policy changes and innovation

**DOI:** 10.1186/s12939-020-01175-7

**Published:** 2020-05-19

**Authors:** Sharon Attipoe-Dorcoo, Rigoberto Delgado, Aditi Gupta, Jennifer Bennet, Nancy E. Oriol, Sachin H. Jain

**Affiliations:** 1 TERSHA LLC, Alpharetta, GA USA; 2 Department of Economics and Finance, University of Texas, El Paso, TX USA; 3 Baylor College of Medicine and Texas Children’s Mobile Clinic Program, Houston, TX USA; 4 Mobile Health Map, Harvard Medical School, Boston, MA USA; 5 The Family Van and Mobile Health Map, Harvard Medical School, Boston, MA USA; 6 Stanford University School of Medicine, Stanford, CA USA

**Keywords:** Mobile clinics, COVID-19 pandemic, Emergency preparedness, Underserved

## Abstract

**Background:**

Mobile Clinics represent an untapped resource for our healthcare system. The COVID-19 pandemic has exacerbated its limitations. Mobile health clinic programs in the US already play important, albeit under-appreciated roles in the healthcare system. They provide access to healthcare especially for displaced or isolated individuals; they offer versatility in the setting of a damaged or inadequate healthcare infrastructure; and, as a longstanding community-based service delivery model, they fill gaps in the healthcare safety-net, reaching social-economically underserved populations in both urban and rural areas. Despite an increasing body of evidence of the unique value of this highly adaptable model of care, mobile clinics are not widely supported. This has resulted in a missed opportunity to deploy mobile clinics during national emergencies such as the COVID-19 pandemic, as well as using these already existing, and trusted programs to overcome barriers to access that are experienced by under-resourced communities.

**Main text:**

In March, the Mobile Healthcare Association and Mobile Health Map, a program of Harvard Medical School’s Family Van, hosted a webinar of over 300 mobile health providers, sharing their experiences, challenges and best practices of responding to COVID 19. They demonstrated the untapped potential of this sector of the healthcare system in responding to healthcare crises. A Call to Action: The flexibility and adaptability of mobile clinics make them ideal partners in responding to pandemics, such as COVID-19. In this commentary we propose three approaches to support further expansion and integration of mobile health clinics into the healthcare system: First, demonstrate the economic contribution of mobile clinics to the healthcare system. Second, expand the number of mobile clinic programs and integrate them into the healthcare infrastructure and emergency preparedness. Third, expand their use of technology to facilitate this integration.

**Conclusions:**

Understanding the economic and social impact that mobile clinics are having in our communities should provide the evidence to justify policies that will enable expansion and optimal integration of mobile clinics into our healthcare delivery system, and help us address current and future health crises.

## Introduction

The COVID-19 pandemic has exacerbated the grave limitations of our health systems when confronting the unexpected emergence of major diseases. As expected, the most affected are the poor [1]. We believe that mobile healthcare delivery programs play an important role in effectively supporting underserved populations during pandemics, and can do so in a cost-effective manner [2]. However, these programs will require additional support and resources, and a significant shift in reimbursement policies.

Currently, an estimated 2000 mobile clinics operate across the United States (US), serving 7 million at-risk people annually [2, 3]. In several mobile clinics in the southern US, the costs of delivering healthcare were lower than the costs of providing care to Medicare beneficiaries in federally funded health centers [4]. Accessibility to displaced or isolated individuals and versatility in the setting of a damaged healthcare infrastructure makes mobile clinics an ideal strategy to provide emergency medical relief [5]. Mobile clinics are a longstanding community-based service delivery model that fills the gaps in healthcare delivery safety-nets, and can also reach social-economically underserved populations in both urban and rural areas [2].

## Context

### Mobile clinic programs and potential during pandemics in the United States

The national Mobile Healthcare Association and the Mobile Health Map, a program of Harvard Medical School’s Family Van, recently collaborated to host a webinar to share best practices in the mobile healthcare sector, gain an understanding of the current state of efforts by the clinics, and exchange how these mobile programs were adapting given the COVID-19 pandemic. A survey was administered to all participants (at least 336 people logged into the meeting, representing about 121 unique mobile programs). While it is unclear whether the survey included a representative sample of mobile clinics, the results demonstrated some of the opportunities, and obstacles mobile clinics face in trying to help communities deal with COVID-19.

Only 19% of surveyed clinics are providing their usual services of care, representing the loss of important community-facing health and wellness programs, and further limiting access to care in at-risk communities. Ten percent of the mobile clinic programs are providing testing to patients in response to COVID-19. For example, the seven mobile clinics associated with the Parkland Health and Hospital System in Dallas, are serving as either drive-through COVID-19 testing sites or triage locations in the parking lots near the emergency department or at regional mega sites. A federally qualified health clinic in Austin, Texas, is also conducting outdoor testing. Nurses triage patients in their vehicles, not on the mobile clinic. Qualified patients then drive around to a doctor to get tested. Cincinnati Children’s is using the mobile clinic to test employees using an algorithm for employees who call a centralized number. The mobile clinic serves as a clean work station that can travel to a satellite location as needed.

**Fig. 1:**
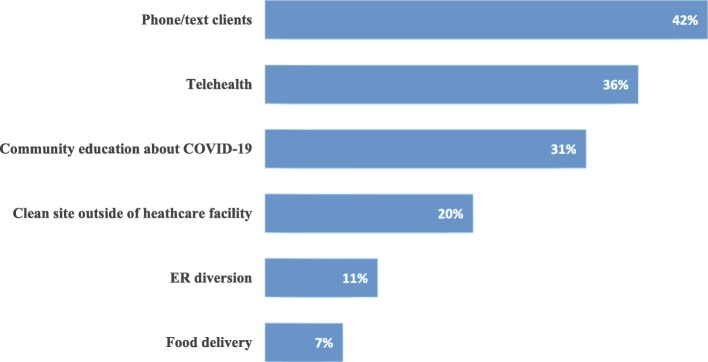
Percent of Mobile Clinic Programs Providing Alternative Services. This graph represents 177 responses from the 121 unique programs in attendance

As illustrated in Fig. 1, all 121 programs have repurposed their operations to serve a variety of needs specific to their patient populations and communities. Many have rapidly adopted telehealth, or are reaching out to their patients via phone calls or texts for wellbeing check-ins “telecare”. For example, the Harvard Medical School’s Family Van is hosting call-in hours, contacting clients directly, and distributing educational material put together by the COVID-19 Health Literacy Project [6]. Morehouse School of Medicine is also converting to a telehealth service, where students will be paired with faculty members, for patients still needing care. A small percent (7%) of mobile clinics are also being used to provide emergency food distribution. The mobile clinics for the Vision to Learn program are now being used to take food and household supplies to seniors in East Los Angeles in a partnership with the Weingart East Los Angeles YMCA, USC Keck, Adventist Health White Memorial, and the American Heart Association. At least one program, in the Philippines, is gathering personal protective equipment from dentists’ offices, and other donors to distribute to first responders.

### Challenges for establishing sustainable and adaptable mobile clinic programs

Despite their ability to adapt rapidly, many mobile clinic programs struggle to sustain annual operations. The largest source of funding for mobile clinics is philanthropy [2, 3], and many will need additional support to respond to this crisis. A small community clinic in Washington D.C., for example, that provides services through the Mexican, Salvadoran, and Guatemalan consulates for most undocumented immigrants and others, is exploring a partnership with George Washington and United Medical Center to use two mobile clinics that have been sitting idle for the past 5 years. Although the Federal Emergency Management Agency (FEMA) deploys mobile medical units during national disasters, these funding efforts happen when the medical system within an area is impacted, and there is an approval time involved in the process [7]. However, if our national preparedness policies related to funding could ensure that mobile clinics are ready and able to respond without the stipulated considerations currently in place for FEMA, particularly given the important expertise of the staff who have demonstrated an ability to reach vulnerable patient populations, then mobile clinics could be a valuable tool in planning for and responding to a wide variety of public health crises. Staff expertise and mobile clinics sitting idle are vital resources needed to be utilized in an all hands on deck approach to address the COVID-19 pandemic.

## Call to Action

### Innovation and policy implications for mobile healthcare delivery: call to action

Although mobile clinics can be an important option for healthcare delivery especially after a disaster has caused stationary facilities to close [8], this model of care has not been widely supported. This has resulted in missed opportunities in our healthcare delivery system. We propose three general approaches to enhance the application of mobile clinics programs and their system-wide integration.

First, we need to recognize the economic contribution mobile clinic programs provide to the healthcare system. The prevention services mobile clinics offer to populations at risk in rural and urban areas provide value to the community in terms of prevented visits to the emergency room, and although economic studies have been completed to quantify such benefits [2], there is considerable more research to be done. There is the need, for example, to research the economic impact of mobile clinics in terms of the triple aim: reduction in per capita average costs of care, benefits to population health, and improvements in patient satisfaction. It is through this lens that we will be able to work with policymakers, providers, and payers to define appropriate reimbursement plans for services provided by mobile clinic programs. This means that we need to move beyond the grant-based model of funding to create sustainable mobile clinic programs.

Second, we believe that specific government funding programs should be implemented to provide needed funding that will allow both the growth and expansion of the number of mobile clinic programs. This will ensure the clinics are readily incorporated into the existing healthcare infrastructure and emergency preparedness. An example of how to implement such a plan is by considering mobile clinics in case management models proposed in the national COVID-19 surveillance system [9]. These models are proposed to increase the capacity of treating patients in isolation facilities, which can include using mobile health clinics through the course of infection, and recovery of patients. These efforts will be successful with enhanced federal reimbursement models that cover community-based resources, as well as state and local health coordination efforts [9]. Establishing these systems now will also strengthen public health and health care systems’ preparedness for future outbreaks.

Third, we propose the creation of national funding programs to expand the use of technology in mobile clinic programs. This will allow mobile clinic programs the opportunity to establish close collaborative involvement with other stakeholders in the healthcare system. For example, reimbursement for the use of telehealth technology in mobile clinics, and the ability to refer and navigate patients in a comprehensive real-time manner. This approach in combination with GIS-based route optimization algorithms could be used to determine priority areas, especially rural areas where stationary facilities are closing at rapid rates [4]. Also, the development of online applications based on data collected through the Mobile Health Map, a program of Harvard Medical School’s Family Van, could be used by state health officials to direct mobile clinic resources to high areas of need. Existing geographic algorithms [4] could be used to determine such locations of need, and the mobile clinics could be used to reach populations efficiently. Additionally, existing measures of the broader range of community needs [2], could help direct mobile clinics to communities in need of prevention services, as well as address the issues of inequity experienced in many under-resourced communities [1].

In conclusion, there is a need to expand our understanding of the economic and social impact of mobile clinics. A clear understanding of the role mobile clinics play in our communities should provide the evidence to justify policies that will enable an optimal integration of mobile clinics into our healthcare delivery system [4]. With national efforts to combat health disparities by addressing social determinants of health, there is now more than ever the need to consider cohesive federal and state funding for mobile health clinics. Although there are current funding efforts for specific populations, such as the homeless populations [3], a comprehensive approach, highlighting the role of mobile clinics in the entire health system, would not only be effective for addressing health outcomes of vulnerable patient populations but will also play a role in contributing to the success of value-based payment models. There are temporary regulatory exceptions that have been made by the Centers for Medicare and Medicaid to reimburse the use of dorm rooms as hospitals to help with efforts to combat the COVID-19 pandemic [10]. This is an example of a need for both innovative and policy-based solutions for this pandemic, as well as future health system strengthening efforts. Mobile healthcare clinics are a vital part of these solutions, and it is time to recognize their broader potential, and include these programs in preparation efforts for current and future health crises.

## Data Availability

The datasets used and/or analyzed during the current study are available from the corresponding author on reasonable request.
